# Neglected but Not Harmless: Characterising Nonantibiotic Antimicrobial Use in Small Animal Practices in Colombia

**DOI:** 10.1002/vms3.71303

**Published:** 2026-10-29

**Authors:** Nathalia M Correa‐Valencia, Susana Arango‐Arcila, Nicolle Kamel Mendoza, Diego Alejandro Sierra‐Zapata, Victoria A. Guzmán‐Mejía, Alejandra Carmona Ramírez, Juan Gonzalo Restrepo Salazar

**Affiliations:** ^1^ CENTAURO Research Group, Faculty of Agricultural Sciences, School of Veterinary Medicine Universidad de Antioquia (UdeA) Medellín Antioquia Colombia; ^2^ Faculty of Agricultural Sciences, School of Veterinary Medicine Universidad de Antioquia (UdeA) Medellín Antioquia Colombia

**Keywords:** antimicrobial stewardship, Colombia, nonantibiotic antimicrobials, One Health, small animal practice, veterinary prescribing

## Abstract

**Background:**

Antimicrobial resistance (AMR) is a growing global threat, yet stewardship efforts have largely overlooked nonantibiotic antimicrobials (NAAs), despite their widespread use and potential role in resistance selection and environmental persistence. Evidence on NAA use in small animal practice, particularly in low‐ and middle‐income countries, remains scarce.

**Objective:**

To characterise patterns of NAA use and assess prescription quality in dogs, cats, and other small animal species treated at a university veterinary hospital in Colombia.

**Methods:**

A retrospective cross‐sectional study was conducted using veterinary prescription records from January to December 2022. A systematic sampling approach (first four prescriptions per day) yielded 1460 prescriptions, of which 731 involved NAAs. Data on animal characteristics, consultation type, and prescription details were extracted, and NAAs were classified by pharmacological group, pharmaceutical form, and route of administration. Bivariate analyses were performed to identify stewardship‐relevant patterns.

**Results:**

NAAs accounted for a substantial proportion of prescriptions, predominantly in dogs (91.5%). Topical antiseptics (49.9%), external antiparasitics (21.3%), and internal antiparasitics (17.1%) were the most frequently used classes. Cutaneous (58.7%) and oral (41.3%) routes predominated, with chlorhexidine as the most common active ingredient (22%). Dermatology consultations were more likely to involve topical antiseptics (55.0% vs. 45.5%; OR = 1.47; 95% CI 1.06–2.02), whereas general consultations showed substantially higher odds of internal antiparasitic use (OR = 5.58; 95% CI 2.93–10.62). While 67.6% of treatments adhered to standard duration, only 38% matched recommended dosing, and key prescription elements were frequently missing, including International Nonproprietary Names (7%), concentration (12.2%), and complete administration instructions (5.5%).

**Conclusions:**

NAAs are widely used in small animal practice but are frequently prescribed with incomplete documentation and suboptimal dosing practices. These findings highlight a critical and underrecognised gap in antimicrobial stewardship and support the need to formally integrate NAAs into surveillance, regulatory frameworks, and stewardship interventions under a One Health approach.

## Graphical abstract

Widely used but poorly documented, nonantibiotic antimicrobials represent an overlooked gap in veterinary practice. Frequent prescribing alongside suboptimal dosing and incomplete records underscores the need to formally include NAAs in antimicrobial stewardship and One Health surveillance efforts.

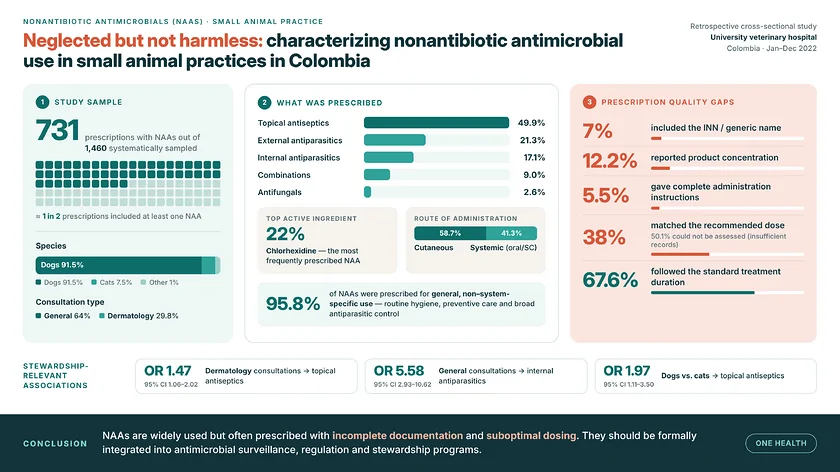

## 1 Introduction

Antimicrobial resistance (AMR) remains a critical global health concern, affecting human, animal, and environmental health and compromising the effectiveness of existing therapies and disease prevention strategies. Although antibiotics (ATBs) have historically been the focus of AMR‐related policies and surveillance, increasing attention is now being directed toward other antimicrobial (AM) agents, particularly nonantibiotic AMs (NAAs), owing to their widespread use and potential contribution to resistance dynamics (Gilbert and McBain 2003; Maillard 2005).

NAAs encompass a broad range of substances with antimicrobial activity, including antiseptics, disinfectants, antifungals, and antivirals. These compounds are commonly used in veterinary settings not only for treatment and infection control but also for hygiene management, wound care, and surgical procedures (Kampf 2018; McDonnell and Russell 1999).

Although NAAs are not classified as ATBs, their subtherapeutic use and environmental persistence may exert selective pressure on microbial communities, potentially promoting cross‐resistance and coselection with ATBs (Buffet‐Bataillon et al. 2012; Maillard 2007).

In recent years, particular concern has arisen over the unregulated and often indiscriminate use of NAAs in both human and veterinary medicine. Studies have reported reduced microbial susceptibility to widely used biocides such as chlorhexidine and benzalkonium chloride, which may compromise the efficacy of critical control measures in clinical practice (Wand et al. 2016; Wesgate et al. 2016). Moreover, NAAs are often used without prescription or dosage standardisation, especially in low‐ and middle‐income countries, where veterinary pharmaceutical oversight may be limited (European Medicines Agency 2021; Paget et al. 2017).

While surveillance efforts have increasingly documented ATB prescription patterns in food‐producing and companion animals, NAAs remain largely unmonitored. This knowledge gap is especially pronounced in regions such as Latin America, where data on veterinary AM use are scarce. In Colombia, for example, national programs have prioritised ATB control in livestock systems, but data on the use of NAAs in companion animals are virtually nonexistent (Instituto Colombiano Agropecuario 2023; World Health Organization 2015).

Understanding the frequency, context, and rationale behind NAA use is essential for developing comprehensive AM stewardship strategies. By exploring patterns of NAA use in this setting, we aim to contribute to a more holistic understanding of AM practices and their implications for AMR control. Therefore, this study aimed to characterise and assess the patterns of NAA use in dogs, cats, and other animal species treated at a university veterinary hospital in Colombia.

## 2 Materials and Methods

### 2.1 Data Collection

An observational cross‐sectional study was carried out to examine the prescription patterns of NAAs in animals treated at a Colombian university veterinary hospital for small animals, following the approach described by Dohoo et al. (2014). The analysis was based on retrospective data extracted from a clinical database that records approximately 10,000 annual admissions. The most common reasons for consultation included routine check‐ups, sample collection for laboratory analysis, preanaesthetic assessments, suture removal, abdominal ultrasound, dermatology visits, radiographs, feline medicine appointments, vaccinations, orthopaedic evaluations, nephrology consultations, and posthospitalisation follow‐ups, collectively accounting for 75% of all visits.

Prescription data were collected for the period from 1 January to 31 December 2022. The dataset included key variables such as consultation date, species, breed, sex, age, weight, affected organ system or diagnosis, and detailed prescription information (active ingredient and trade name, pharmaceutical form, dosage, treatment interval and duration, and route of administration). For consistency and representativeness, the first four prescriptions recorded each day during the study period were selected. On days with fewer than four prescriptions, the shortfall was compensated by selecting prescriptions from the following day. This approach yielded a total of 1460 veterinary prescription records. Because the sampling unit was the prescription rather than the individual patient, the resulting NAA dataset reflects the relative frequency of NAA use among all dispensed prescriptions, but it does not allow direct comparison with the underlying demographic distribution of the entire hospital caseload, which was not extracted in this study. The hospital is known to predominantly serve canine patients, with a relatively small proportion of feline and other small animal species, which is consistent with the species distribution observed in our sample.

This study was reported in accordance with the STROBE‐Vet guidelines for observational veterinary studies.

### 2.2 Database Management and Product Categorisation

To support structured analysis, the recorded prescriptions were standardised and categorised using multiple criteria. Each NAA product was identified and classified according to its active ingredient, therapeutic indication, and pharmaceutical formulation. Given the lack of a universal classification system for NAAs, the active ingredients were grouped into broader pharmacological classes based on their primary function (e.g., antiseptics, external antiparasitics, anticestodals, antifungals, and combined formulations). Compounds such as acids, quaternary ammonium derivatives, and plant‐based antiseptics were grouped under topical antiseptics when relevant. Products were also classified by pharmaceutical form (e.g., tablet, topical solution, shampoo), route of administration (cutaneous, oral, otic, etc.), and target organic system. The ‘general use’ category was defined operationally as prescriptions intended for broad‐spectrum or non–system‐specific applications, including topical antiseptics applied to the skin or ear canal as part of routine hygiene or preventive care, external antiparasitics for whole‐body ectoparasite control, and broad‐spectrum endoparasiticides used for general antiparasitic prophylaxis. Conversely, prescriptions targeting a specific anatomical system (e.g., integumentary lesions, urinary tract, gastrointestinal tract, ocular structures) were classified under their respective categories. For the purpose of cross‐tabulation and comparative analyses, several variables were further consolidated into broader, mutually exclusive categories: route of administration was grouped into topical (cutaneous, otic, ophthalmic, inhaled) versus systemic (oral, subcutaneous); pharmaceutical form was grouped into cutaneous topical, oral solid, oral liquid, ophthalmic topical, and injectable; and the original 12 pharmacological subgroups were collapsed into five broader classes (topical antiseptics, external antiparasitics, internal antiparasitics, antifungals, and combination therapies). Active ingredients reported with and without their International Nonproprietary Name (INN) were merged for analytical purposes (e.g., chlorhexidine entries, irrespective of INN reporting). The consolidated categories are presented in Table 1, while the full ungrouped detail is provided in Table S1. Data entry was conducted via Microsoft Excel 2013 (Microsoft Corporation, Redmond, WA, USA).

**TABLE 1:** Consolidated descriptive results for nonantibiotic antimicrobial prescriptions in animals attending the small animal veterinary hospital of study (Antioquia, Colombia), 2022. A detailed, ungrouped breakdown of all the variables and individual active ingredients is provided in Table S1.

Variable	Category	Frequency (%)
**Animal species**	Dogs	669 (91.5)
	Cats	55 (7.5)
	Other small animal species	7 (1)
**Type of consultation**	General	468 (64)
	Specialist—dermatology	218 (29.8)
	Specialist—other (nephrology, endocrinology, imaging, ophthalmology, dentistry, preanaesthetic)	23 (3.1)
	Follow‐up	19 (2.6)
	Hospitalisation	3 (0.4)
**Pharmaceutical form**	Cutaneous topical (solution, shampoo, wipe, cream, spray, soap, ointment, salve, collar)	429 (58.7)
	Oral solid (tablet, capsule)	247 (33.8)
	Oral liquid (suspension, oral solution)	47 (6.4)
	Ophthalmic topical (eye drops)	7 (1)
	Injectable	1 (0.1)
**Route of administration**	Topical (cutaneous, otic, ophthalmic, inhaled)	429 (58.7)
	Systemic (oral, subcutaneous)	302 (41.3)
**Pharmacological class**	Topical antiseptics	365 (49.9)
	External antiparasitics	156 (21.3)
	Internal antiparasitics (anticestodal, antinematodal, anticoccidial, antiprotozoal)	125 (17.1)
	Combination therapies	66 (9)
	Antifungals	19 (2.6)
**Target organic system**	General^a^	700 (95.8)
	Integumentary^b^	17 (2.3)
	Urinary	8 (1.1)
	Gastrointestinal	5 (0.6)
	Ocular	1 (0.2)
**Most frequent active ingredients** ^c^	Chlorhexidine (alone or in combinations)	258 (35.3)
	Lactic acid + acetylsalicylic acid	98 (13.4)
	Fenbendazole + pyrantel + praziquantel	80 (10.9)
	Fluralaner	70 (9.6)
	Afoxolaner (alone or with milbemycin)	59 (8.1)
	Metronidazole	25 (3.4)
	Other active ingredients (full list in Table S1)	141 (19.3)

^a^ ‘General’ refers to prescriptions intended for broad‐spectrum or non–system‐specific applications (topical antiseptics for routine skin/ear hygiene, external antiparasitics for whole‐body ectoparasite control, and broad‐spectrum endoparasiticides used for general antiparasitic prophylaxis).

^b^ Includes skin, ear canal, and oral cavity.

^c^ Active ingredients reported with and without their International Nonproprietary Name (INN; Syn. generic name) were merged for this consolidated table; the full list of all individual entries (including all single‐prescription items) is available in Table S1.

### 2.3 Statistical Analysis

Categorical variables are summarised as absolute and relative frequencies. To examine associations relevant to antimicrobial stewardship targeting, bivariate comparisons were performed between (i) animal species (dogs vs. cats; the ‘other small animal species’ group was excluded from inferential tests because of its small sample size [*n* = 7]) and key prescribing outcomes (route of administration and pharmacological class) and between (ii) consultation type (general vs. specialist–dermatology) and the same outcomes. Pearson's chi‐square test was used for 2 × 2 contingency tables, with Fisher's exact test substituted when expected cell counts were ≤ 5. Odds ratios (ORs) with 95% confidence intervals (CIs) were calculated as effect size measures, applying Haldane's correction (0.5 added to all cells) when zero cells were present. A two‐sided *p* value < 0.05 was considered to indicate statistical significance. All analyses were performed in Stata v.19.5 (StataCorp, 2025, College Station, TX, USA).

## 3 Results

Table 1 provides a descriptive overview of NAA prescriptions recorded at the university small animal veterinary hospital over a one‐year period. It summarises the distribution of NAA use by animal species, type of consultation, pharmaceutical form, route of administration, target organic system, and active ingredient. Most prescriptions involved a limited number of commonly used NAAs; however, a diverse set of less frequent active ingredients—each representing a single prescription—were grouped under the category ‘Others’.

A total of 731 cases involving the use of NAAs were recorded from the total number of veterinary prescriptions. Most were prescribed to dogs (91.5%), followed by cats (7.5%), and a small proportion were prescribed to other small animal species, such as rabbits, parrots, cockatoos, hamsters, and pigs (1%). General consultations were the most frequent (64%), whereas specialist dermatology consultations accounted for 29.8% of the cases.

When grouped into broader pharmacological classes (see Section 2 and Table 1), nearly half of the prescriptions corresponded to topical antiseptics (*n* = 365; 49.9%), followed by external antiparasitics (*n* = 156; 21.3%), internal antiparasitics (*n* = 125; 17.1%; encompassing anticestodal, antinematodal, anticoccidial, and antiprotozoal agents), combination therapies (*n* = 66; 9.0%; encompassing topical antiseptic–antifungal, antibacterial–antifungal, antiprotozoal–antibacterial, and anticestodal–anticoccidial combinations), and antifungals as a stand‐alone class (*n* = 19; 2.6%). A full ungrouped breakdown of the original 12 pharmacological subgroups is provided in Table S1.

A clinical diagnosis or reason for consultation was documented in 96.4% (*n* = 705) of the records, whereas in 3.6% (*n* = 26), it was reported as absent or undefined. The most frequent indications were dermatological conditions—including canine atopy (*n* = 59), superficial pyoderma (*n* = 41), atopic dermatitis (*n* = 28), food allergy (*n* = 26), flea‐bite allergic dermatitis (*n* = 22), allergic contact dermatitis (*n* = 21), and external otitis (*n* = 21)—followed by intestinal parasitism (*n* = 32 across specific and unspecified entries) and giardiasis (*n* = 12). Notably, 94 prescriptions (12.9%) were issued during routine general medical examinations without a specific pathological diagnosis, suggesting prophylactic or preventive use, particularly for external and internal antiparasitics and topical antiseptics for routine hygiene.

When pharmaceutical forms were consolidated into broader categories, cutaneous topical preparations (including topical solutions, shampoos, wipes, creams, sprays, soaps, ointments, salves, and collars) predominated (*n* = 429; 58.7%), followed by oral solid forms (tablets and capsules; *n* = 247; 33.8%), oral liquid forms (suspensions and oral solutions; *n* = 47; 6.4%), ophthalmic topical forms (eye drops; *n* = 7; 1.0%), and injectable forms (*n* = 1; 0.1%). Consistently, the route of administration was topical (cutaneous, otic, ophthalmic, or inhaled) in 58.7% of prescriptions (*n* = 429) and systemic (oral or subcutaneous) in 41.3% (*n* = 302). A detailed breakdown of all 16 individual pharmaceutical‐form categories and 6 administration routes is reported in Table S1.

In species‐specific terms, dogs were predominantly prescribed topical solutions (*n* = 204), tablets (*n* = 232), and shampoos (*n* = 137). Other formulations included suspensions (*n* = 36), wipes (*n* = 28), creams (*n* = 10), and capsules (*n* = 6), with fewer cases involving ointments, injectable or oral solutions, and collars. In cats, the most common forms were topical solutions (*n* = 31) and tablets (*n* = 9), followed by eye drops (*n* = 6) and suspensions (*n* = 6). For other small animals, only a few prescriptions were recorded, mainly topical solutions (*n* = 4), oral solutions (*n* = 2), and suspensions (*n* = 1). With respect to the target organic system, NAAs were most commonly prescribed for general use (*n* = 700; 95.8%), predominantly topical antiseptics for routine skin or ear hygiene, external antiparasitics, and broad‐spectrum endoparasiticides, followed by skin (2.3%), urinary tract (1.1%), digestive tract (0.6%), and ocular structures (0.2%).

Among the active ingredients, chlorhexidine was the most frequently prescribed (22%), followed by lactic acid‐based combinations and broad‐spectrum antiparasitics such as fenbendazole–pyrantel–praziquantel.

In terms of treatment duration, most prescriptions (67.6%) were consistent with standard recommendations (Restrepo 2021). In 21.5% of the patients, the duration could not be determined based on available medical records, while 9.6% of the treatments were longer than the standard, and 1.4% were shorter. With respect to dose equivalence, approximately half of the prescriptions (50.1%) could not be assessed because of insufficient documentation. Among those evaluated, 38% were consistent with the recommended dose (Restrepo 2021), 4.9% were underdosed, 4.4% were overdosed, and 2.6% were deemed not applicable. With respect to the dosing interval, most treatments (82.9%) adhered to the standard interval (Restrepo 2021), while 11.6% exceeded it, 0.8% were shorter, and 4.7% could not be assessed.

Table 2 summarises the presence or absence of key elements in the recorded veterinary prescriptions for NAAs. Each element was assessed as either present (Yes) or absent (No), and the results are presented as absolute numbers with percentages. Such an assessment revealed substantial variability in the inclusion of key elements. Nearly all prescriptions reported the trade name of the product (99.7%), yet only 7% included the INN or generic name, and only 2.6% included both identifiers. With respect to pharmaceutical specifications, the pharmaceutical form was present in slightly more than half of the prescriptions (52.1%), whereas the concentration and presentation of the product were reported in only 12.2 and 0.8% of the cases, respectively. Elements related to dosage clarity, such as quantity in numeric form (4.7%), quantity written (3.3%), and complete administration instructions (5.5%), were rarely documented.

**TABLE 2:** Completeness of key prescription elements in nonantibiotic antimicrobial records from a university small animal veterinary hospital (Antioquia, Colombia), 2022.

Prescription element	Yes (*n*, %)	No (*n*, %)
Trade name included in the prescription	729 (99.7)	2 (0.3)
INN included in the prescription	51 (7)	680 (93)
Both trade name and INN included	19 (2.6	712 (97.4)
Pharmaceutical form included	381 (52.1)	350 (47.9)
Concentration included	89 (12.2)	642 (87.8)
Pharmaceutical presentation included	6 (0.8)	725 (99.2)
Quantity (numeric) included	553 (75.6)	178 (24.4)
Quantity (written out) included	4 (0.5)	727 (99.5)
Complete administration instructions included	403 (55.1)	328 (44.9)

Abbreviations: INN = International nonproprietary name (Syn. generic name).

To explore prescribing patterns relevant to stewardship targeting, we examined the associations between species and consultation type with key outcomes (Table 3). When dogs and cats were compared, topical antiseptics were significantly more frequently prescribed in dogs (51.0% vs. 34.5%; OR = 1.97; 95% CI 1.11–3.50; *p* = 0.028), whereas internal antiparasitics tended to be more frequent in cats (27.3% vs. 16.1%; OR = 0.51; 95% CI 0.27–0.96; *p* = 0.054). The external antiparasitics, combination therapies, and overall topical vs. systemic distribution did not differ significantly between species. Stand‐alone antifungals were prescribed exclusively to dogs (*n* = 19), with no antifungal prescriptions recorded for cats during the study period (Fisher's exact *p* = 0.39). When general and specialist–dermatology consultations were compared, dermatology consultations were significantly more likely to involve topical antiseptics (55.0% vs. 45.5%; OR = 1.47; 95% CI 1.06–2.02; *p* = 0.025) and the topical route of administration (72.5% vs. 51.9%; OR = 2.44; 95% CI 1.72–3.45; *p* < 0.001), whereas general consultations were more than five times more likely than dermatology consultations to involve internal antiparasitics (22.9% vs. 5.0%; OR = 5.58; 95% CI 2.93–10.62; *p* < 0.001). The overall association between route of administration and pharmacological class was highly significant (chi^2^ = 465.3, *p* < 0.001), reflecting that topical antiseptics, external antiparasitics, and most combinations were almost exclusively administered topically, whereas internal antiparasitics were administered systemically orally.

**TABLE 3:** Bivariate associations between animal species and consultation type with key nonantibiotic antimicrobial prescribing outcomes (Antioquia, Colombia, 2022).

Comparison	Outcome	Group A (*n*/*N*, %)	Group B (*n*/*N*, %)	OR (95% CI)	*p* value
Dogs vs. cats	Topical antiseptics	341/669 (51)	19/55 (34.5)	1.97 (1.11–3.50)	0.028
Dogs vs. cats	External antiparasitics	143/669 (21.4)	13/55 (23.6)	0.88 (0.46–1.68)	0.825
Dogs vs. cats	Internal antiparasitics	108/669 (16.1)	15/55 (27.3)	0.51 (0.27–0.96)	0.054
Dogs vs. cats	Combination therapies	58/669 (8.7)	8/55 (14.5)	0.56 (0.25–1.24)	0.226
Dogs vs. cats	Antifungals (alone)	19/669 (2.8)	0/55 (–)	Not computable^a^	0.388^b^
Dogs vs. cats	Topical route	386/669 (57.7)	38/55 (69.1)	0.61 (0.34–1.10)	0.132
Dermatology vs. general	Topical antiseptics	120/218 (55)	213/468 (45.5)	1.47 (1.06–2.02)	0.025
Dermatology vs. general	External antiparasitics	50/218 (22.9)	105/468 (22.4)	1.03 (0.70–1.51)	0.962
Dermatology vs. general	Internal antiparasitics^c^	11/218 (5)	107/468 (22.9)	0.18 (0.09–0.34)	< 0.001
Dermatology vs. general	Topical route	158/218 (72.5)	243/468 (51.9)	2.44 (1.72–3.45)	< 0.001

*Note*: For each row, ‘Group A’ corresponds to the first group named in the ‘Comparison’ column and ‘Group B’ to the second; the OR is reported as the odds in Group A relative to Group B. Pearson's chi‐square test was used for 2 × 2 contingency tables; Fisher's exact test was used when expected cell counts were ≤ 5. Owing to its small sample size, the ‘other small animal species’ group (*n* = 7) was excluded from the inferential analyses.

^a^ Owing to zero cells (no antifungal prescriptions in cats), the OR cannot be reliably estimated.

^b^ Fisher's exact test.

^c^ For the dermatology vs. general comparison of internal antiparasitics, an OR < 1 is consistent with internal antiparasitics being substantially less common in dermatology consultations than in general consultations (the equivalent OR for general vs. dermatology is 5.58; 95% CI 2.93–10.62).

OR = odds ratio; CI = confidence interval.

## 4 Discussion

This study aimed to characterise the use patterns of NAAs in animals treated at a university small animal veterinary hospital in Colombia. While NAAs are often overlooked in AMR surveillance, our findings demonstrate that they are widely prescribed in veterinary clinical settings, often with limited regulatory oversight or documentation standards. The results highlight the predominance of a few key agents, such as chlorhexidine and broad‐spectrum antiparasitics, as well as significant gaps in prescription completeness and standardisation.

The frequent use of topical antiseptics and antiparasitic agents in this study aligns with the findings of previous research, which suggests that NAAs are widely employed in dermatological and preventive care in companion animals (Kampf 2018; McDonnell and Russell 1999). Notably, chlorhexidine was the most prescribed NAA, which is consistent with global trends in both human and veterinary medicine (Kampf 2016). However, this widespread use raises concerns regarding bacterial resistance. Studies have demonstrated reduced susceptibility to chlorhexidine among clinical isolates, including methicillin‐resistant *Staphylococcus aureus* (MRSA), raising concerns about cross‐resistance between biocides and antibiotics (Maillard 2005; Wand et al. 2016).

The use of antiparasitics such as fluralaner, afoxolaner, and fenbendazole–pyrantel–praziquantel combinations is also notable. These compounds are essential for controlling ecto‐ and endoparasites in companion animals, but their overuse may contribute to resistance in parasites of veterinary and zoonotic importance. Importantly, many of these molecules have close analogues or identical uses in human medicine, suggesting the potential for resistance spillovers across species. For example, metronidazole and antifungals such as miconazole and ketoconazole are used in both human and veterinary medicine, making prudent use vital to preserve efficacy across sectors.

From a One Health perspective, the implications of widespread and sometimes undocumented NAA use are concerning. The environmental persistence of compounds such as chlorhexidine (Kampf 2019; McDonnell and Russell 1999) and imidacloprid (Bonmatin et al. 2015; Morrissey et al. 2015; Wesgate et al. 2016; Wood and Goulson 2017) has been documented in wastewater and agricultural runoff, posing risks to aquatic ecosystems and wildlife. Additionally, the potential for toxicity in humans, especially among pet owners exposed to residual products, cannot be overlooked. Misuse or overuse of NAAs may also lead to the emergence of resistant microbial strains in household environments, increasing the risk of zoonotic transmission.

Moreover, our results revealed major gaps in the completeness of veterinary prescriptions. While trade names were almost universally reported (99.7%), the inclusion of generic names (7%), concentration (12.2%), and complete administration instructions (5.5%) was very low. Such documentation deficiencies not only compromise clinical reproducibility but also may hinder efforts to implement effective antimicrobial stewardship strategies (Paget et al. 2017). Inconsistent reporting impedes risk assessment, pharmacovigilance, and rational drug use monitoring.

The comparative analyses provide preliminary evidence with direct implications for stewardship targeting. The greater odds of topical antiseptic use in dogs than in cats and in dermatology consultations than in general consultations suggest that interventions aimed at rationalising chlorhexidine and related antiseptics—including dosing standardisation, prescription completeness and prevention of unsupervised owner‐driven use—would have the highest yield if they focused on canine dermatology workflows. Conversely, the substantially higher odds of internal antiparasitic prescriptions in general consultations than in dermatology consultations point to general‐practice deworming protocols as a natural target for stewardship interventions on broad‐spectrum antiparasitics, particularly considering that several of these products are widely available over the counter and may be administered without a specific parasitological diagnosis. The trend towards higher proportional use of internal antiparasitics in cats, although only borderline significant, warrants confirmation in larger feline‐enriched samples. Taken together, these patterns illustrate how route‐, species‐, and service‐stratified analyses can help prioritise where stewardship effort is most likely to be impactful within a busy small‐animal hospital.

These findings emphasise the urgent need for veterinary‐specific antimicrobial stewardship programs (ASPs) that extend beyond antibiotics. In this context, the concept of ‘antiseptic stewardship’ has been proposed to promote the rational use of biocides, encouraging the same level of scrutiny applied to ATBs (Kampf 2018; Maillard 2018). The integration of NAAs into national AMR surveillance programs, such as those recommended by the World Organization for Animal Health (2024), would support a more comprehensive approach to resistance mitigation.

This study presents several notable strengths. Foremost is its novel focus on NAAs in companion animal medicine—an area that remains largely overlooked in AMR research. By analysing data from a university veterinary hospital, the study benefits from access to high‐quality clinical records, ensuring both data accuracy and contextual relevance. Furthermore, the dataset captured a wide spectrum of NAA formulations marketed in Colombia, including products that are routinely available over the counter (OTC) in veterinary pharmacies and agro‐veterinary outlets—such as chlorhexidine‐based antiseptics, broad‐spectrum endoparasiticides (e.g., fenbendazole–pyrantel–praziquantel combinations), and several topical antifungal–antiseptic combinations—alongside products typically dispensed under veterinary prescription, such as isoxazoline‐class ectoparasiticides (fluralaner, afoxolaner, and sarolaner). However, a systematic product‐by‐product classification of regulatory status (OTC vs. veterinary‐prescription‐only vs. compounded) was not performed in this study and is identified below as a limitation. This comprehensive approach offers valuable evidence to inform regulatory frameworks, veterinary curricula, and antimicrobial stewardship programs, especially in underrepresented regions such as Latin America. However, several limitations must be acknowledged. First, the retrospective nature of the study limited our ability to assess clinical rationale and treatment outcomes. Second, prescription records were frequently incomplete, which hindered the consistent evaluation of key variables such as treatment duration, dosing interval, and concentration. Although a clinical diagnosis was documented in the majority of records (96.4%), the specific indication for each NAA prescription (e.g., empirical treatment, preventive use, perioperative antisepsis) was not consistently differentiated from the underlying diagnosis, limiting our ability to evaluate the prescribing rationale at a finer granularity. Additionally, the data were derived from a systematic sample (the first four prescriptions recorded each day), rather than the full hospital caseload. While this approach allowed for a feasible and replicable method of data collection over the calendar year, it may have underestimated the actual frequency and diversity of NAA use. The study was conducted in a single university veterinary hospital, whose patient demographics and prescribing practices—dominated by canine consultations—may not reflect those of private, rural, or other institutional settings across Colombia. Thus, while the findings offer a valuable snapshot of current prescription patterns, their generalisability at the national level remains limited. Moreover, inconsistencies in record‐keeping highlight the urgent need to improve clinical documentation and digital data management to support future monitoring and antimicrobial stewardship efforts. An additional consideration is that comparative denominator data—namely, the species‐ and service‐level distribution of the entire hospital caseload during the study period—were not extracted; therefore, we cannot formally assess whether NAAs were over‐ or underrepresented in any particular species or clinical service relative to the general patient population. Future studies should incorporate hospital‐wide caseload denominators to enable such comparative inferences and to identify priority targets for stewardship interventions. In addition, although the dataset captured products with varied regulatory profiles, a formal product‐by‐product classification of regulatory status (OTC, veterinary‐prescription‐only, or compounded) was not performed retrospectively, as this information was not consistently recorded in the medical chart and would have required cross‐referencing each commercial product with national regulatory databases (e.g., INVIMA registries in Colombia). This is a relevant limitation given that OTC NAAs may be more prone to misuse—owners can access them without veterinary consultation, potentially leading to inappropriate dosing, prolonged exposure, and unsupervised use, all of which are recognised drivers of resistance selection. Future studies should incorporate explicit OTC/prescription/compounded categorisation to better characterise exposure pathways and inform targeted stewardship and regulatory interventions. Multicentre studies with broader geographic and institutional representation are recommended to validate and expand upon these findings.

## 5 Conclusion

This study highlights the high frequency and diversity of NAA use in small animal practice and reveals important gaps in prescription quality and documentation. Comparative analyses further revealed that prescribing patterns differ meaningfully by species and by consultation type, identifying canine dermatology workflows and general‐practice deworming as concrete priority targets for future stewardship interventions. Given the potential for environmental contamination, resistance development, and human health impacts, NAAs should be integrated into antimicrobial stewardship and regulatory frameworks. These findings support calls for broader One Health approaches that address all classes of antimicrobials—not just antibiotics, in both veterinary and human medicine.

## Supplementary Material

Additional supporting information may be found online in the Supporting Information section. **Table** **S1**. Detailed (ungrouped) descriptive results for nonantibiotic antimicrobial prescriptions in animals attending the small animal veterinary hospital of study (Antioquia, Colombia), 2022, including the full breakdown of all individual active ingredients (with and without their International Nonproprietary Name) and all original pharmaceutical‐form, route, and consultation‐type categories.

## Author Contributions

Nathalia M. Correa‐Valencia: methodology, data curation, formal analysis, Writing – original draft, Writing – review & editing. Susana Arango‐Arcila, Nicolle Kamel Mendoza, Diego Alejandro Sierra‐Zapata, Victoria A. Guzmán‐Mejía, and Alejandra Carmona Ramírez: investigation, data curation, Writing – review & editing. Juan Gonzalo Restrepo Salazar: Conceptualisation, methodology, supervision, Writing – review & editing. All authors read and approved the final version of the manuscript.

## Funding

The authors have nothing to report.

## Ethics Statement

This study did not involve any animal experimentation, as it relied solely on data collected during routine clinical consultations at a university small veterinary hospital. As such, approval from an animal ethics committee was not needed.

## Conflicts of Interest

None of the authors of this article have a financial or personal relationship with other people or organisations that could inappropriately influence or bias the content of the paper.

## Supporting information




**Supporting File 1**: vms371303‐sup‐0001‐S1.pdf

## Data Availability

The data that support the findings of this study are available from the corresponding author upon reasonable request.
