# Supplementary material for: Neglected but Not Harmless: Characterising Nonantibiotic Antimicrobial Use in Small Animal Practices in Colombia
Source: Vet Med Sci. 2026 Oct 29;12(6):e71303. doi: 10.1002/vms3.71303 (PMC13720525; doi:10.1002/vms3.71303)
Supplement: Supplementary file 1 [file VMS3-12-e71303-s001.pdf]

Supplementary Material

Manuscript: "Neglected but not Harmless: Characterizing Nonantibiotic Antimicrobial Use in Small Animal Practices in Colombia"

Table S1. Detailed descriptive results for nonantibiotic antimicrobial prescriptions in animals attending the small animal veterinary hospital of study (Antioquia, Colombia), 2022. This table provides the full granular breakdown of variables that were consolidated into broader categories in Table 1 of the main manuscript, including all individual active ingredients (with and without their International Nonproprietary Name [INN]) and all original pharmaceutical-form, route, and consultation-type categories.

Variable	Category	Frequency (%)
Animal species	Dogs	669 (91.5)
	Cats	55 (7.5)
	Other small animal species	7 (1)
Type of consultation	General	468 (64)
	Specialist – Dermatology	218 (29.8)
	Follow-up	19 (2.6)
	Specialist – Nephrology	11 (1.5)
	Specialist – Endocrinology	4 (0.5)
	Hospitalization	3 (0.4)
	Specialist – Imaging	3 (0.4)
	Specialist – Ophthalmology	2 (0.3)
	Specialist – Dentistry	2 (0.3)
	Preanaesthetic consultation	1 (0.2)
Pharmaceutical form	Tablet	241 (33)
	Topical solution	239 (32.7)
	Shampoo	137 (18.7)
	Suspension	43 (5.9)
	Wipe	30 (4.1)

	Cream	11 (1.4)
	Eye drops	7 (1)
	Capsule	6 (0.7)
	Oral solution	4 (0.5)
	Spray	3 (0.4)
	Soap	3 (0.4)
	Liquid soap	2 (0.3)
	Ointment	2 (0.3)
	Collar	1 (0.2)
	Salve	1 (0.2)
	Injectable solution	1 (0.2)
Route of administration	Cutaneous	316 (43.2)
	Oral	301 (41.2)
	Otic	104 (14.1)
	Ophthalmic	7 (1)
	Inhaled	2 (0.3)
	Subcutaneous	1 (0.2)
Target organic system	General	700 (95.8)
	Integumentary*	17 (2.3)
	Urinary	8 (1.1)
	Gastrointestinal	5 (0.6)
	Ocular	1 (0.2)
Active ingredient	Chlorhexidine (without INN)	161 (22)
	Lactic acid, acetylsalicylic acid (without INN)	95 (13)
	Fenbendazole, pyrantel, praziquantel (without INN)	79 (10.8)
	Fluralaner (without INN)	70 (9.6)
	Chlorhexidine, miconazole (without INN)	38 (5.2)
	Afoxolaner (without INN)	29 (4)
	Chlorhexidine	27 (3.7)

Afoxolaner, milbemycin (without INN)	25 (3.4)
Chlorhexidine, aloe vera (without INN)	24 (3.3)
Metronidazole	19 (2.6)
Imidacloprid, moxidectin (without INN)	10 (1.4)
Chlorhexidine, cetrimide	9 (1.2)
Miconazole (without INN)	8 (1.1)
Selamectin (without INN)	8 (1.1)
Cranberry, collagen, polydextrose, vitamin C, sucralose, sodium benzoate and citrate (without INN)	8 (1.1)
Orbifloxacin, posaconazole, mometasone (without INN)	7 (1)
Boric acid, borate and sodium chloride, EDTA, sorbic acid (without INN)	6 (0.7)
Metronidazole (without INN)	6 (0.7)
Hypochlorous acid (without INN)	6 (0.7)
Sarolaner, pyrantel, moxidectin (without INN)	5 (0.6)
Sarolaner (without INN)	4 (0.5)
Fenbendazole, praziquantel, diclazuril (without INN)	4 (0.5)
Pyrantel, praziquantel (without INN)	4 (0.5)
Fenbendazole	4 (0.5)
Praziquantel, milbemycin (without INN)	4 (0.5)
Afoxolaner, milbemycin	4 (0.5)
Ketoconazole, gentamicin, dexamethasone (without INN)	3 (0.4)
Lactic acid, acetylsalicylic acid	3 (0.4)
Ketoconazole (without INN)	3 (0.4)
Nitrofurazone (without INN)	3 (0.4)
Toltrazuril	3 (0.4)
Fipronil (without INN)	3 (0.4)
Fenbendazole, praziquantel, toltrazuril (without INN)	3 (0.4)
Fenbendazole, toltrazuril (without INN)	3 (0.4)

Fipronil, methoprene, praziquantel, eprinomectin (without INN)	2 (0.3)
Zinc oxide (without INN)	2 (0.3)
Lauryl alcohol, cocamidopropyl betaine, cocamide MEA, plant extracts and vitamins (without INN)	2 (0.3)
Glycerin, calcium carbonate, carrageenan, sodium lauryl sulfate (without INN)	2 (0.3)
Praziquantel, febantel, pyrantel (without INN)	2 (0.3)
Benzyl benzoate, pine oil (without INN)	2 (0.3)
Benzoyl (without INN)	2 (0.3)
Albendazole, praziquantel (without INN)	2 (0.3)
Others**	30 (4.1)

*Includes skin, ear canal, and oral cavity.

**Includes active ingredients that were each prescribed only once during the study period (full list available upon request from the corresponding author).

EDTA = Ethylenediaminetetraacetic acid; INN = International Nonproprietary Name (Syn. generic name); MEA = Monoethanolamine salt of coconut fatty acids.
